# The *in vivo* and *in vitro* corrosion behavior of MgO/Mg-Zn-Ca composite with different Zn/Ca ratio

**DOI:** 10.3389/fbioe.2023.1222722

**Published:** 2023-06-22

**Authors:** Shuquan Zhang, Chaokun Tang, Jiangtao Feng, Qi Wang, Chenguang Li, Weihao Zhang, Fengxin Zhou, Feng Xue, Baoshan Xu, Shaoyuan Lyu, Minfang Chen, Hao Wang

**Affiliations:** ^1^ Tianjin Key Laboratory of Brain Science and Neural Engineering, Academy of Medical Engineering and Translational Medicine, Tianjin University, Tianjin, China; ^2^ School of Materials Science and Engineering, Tianjin University of Technology, Tianjin, China; ^3^ Department of Orthopedics, Integrated Chinese and Western Medicine Hospital, Tianjin University, Tianjin, China; ^4^ National Demonstration Center for Experimental Function Materials Education, Tianjin, China; ^5^ Key Laboratory of Display Materials and Photoelectric Device (Ministry of Education), Tianjin, China

**Keywords:** MgO/Mg-Zn-Ca composite, *in vivo* corrosion, *in vitro* corrosion, second phase corrosion, bone growth

## Abstract

The effect of Zn/Ca ratio on the corrosion behavior of Mg–3Zn-0.2Ca-1.0MgO (3ZX) and Mg–1Zn-0.2Ca-1.0MgO (ZX) was investigated on the as-extruded specimens. Microstructure observations revealed that the low Zn/Ca ratio led to the grain growth from 1.6 µm in 3ZX to 8.1 µm in ZX. At the same time, the low Zn/Ca ratio changed the nature of second phase from the existence of Mg-Zn and Ca_2_Mg_6_Zn_3_ phases in 3ZX to the dominated Ca_2_Mg_6_Zn_3_ phase in ZX. The local galvanic corrosion caused by the excessive potential difference was alleviated obviously due to the missing of MgZn phase in ZX. Besides, the *in vivo* experiment also showed that ZX composite exhibited a good corrosion performance and the bone tissue around the implant grew well.

## 1 Introduction

Magnesium (Mg) possesses numerous advantages for medical applications within the human body, including high specific strength, low density, and a close Young’s modulus with bone (Tsakiris et al., 2021). At the same time, as an essential element of human metabolism, Mg is biocompatible and bioresorbable (Paul et al., 2020). Moreover, Mg demonstrates biodegradability through its spontaneous dissolution in aqueous media (Song and Atrens, 2003). These properties give Mg alloys a great potential for application in the field of biodegradable implants. However, the major limitations, including high hydrogen precipitation during degradation and unpredictable degradation rates, prevent the widespread application of Mg alloys (Peng et al., 2022).

Alloying is considered to be one of the most important ways to improve corrosion properties of Mg. The second phases in the Mg matrix through adding various alloying elements have a significant influence on the corrosion behavior of Mg alloys (Zhang et al., 2012; Zeng et al., 2015). On the one hand, the morphology and structure of the second phase play key role during corrosion process (Lu et al., 2015; Zander and Zumdick, 2015). The fine and diffuse second-phase particles promotes the formation of continuous corrosion product films (Zhao et al., 2008; Cai et al., 2012), whereas a continuous and reticulated particles can serve as a physical barrier against corrosion diffusion, consequently enhancing the corrosion resistance of Mg alloys (Wang et al., 2011; Cubides et al., 2020). However, a separate and coarser second phase can lead to localized microelectron corrosion, producing severe localized corrosion (Zhang et al., 2020). On the other hand, the nature of the second phases also influences the corrosion behavior of Mg alloys through the formation of various compounds (Bakhsheshi-Rad et al., 2012; Jin et al., 2020). The greater the potential difference between the second phase and the matrix, the more severe the microelectron corrosion present (Liu et al., 2019). Generally, Mg matrix exhibits a negative potential compared to the second phase particles, resulting in the Mg matrix acting as an anode for micro-electro-coupling and undergoing preferential corrosion (Esmaily et al., 2017).

Therefore, the selection of biosafe alloying elements and the minimization of the potential difference between the second phase and the matrix became the key point in the design of biomedical Mg alloys. Although the addition of Al, Zr and rare earth elements (REEs) can effectively improve the corrosion resistance of Mg alloys (Jung et al., 2015; Sun et al., 2022; Chen et al., 2023), but excessive intake of Al may cause Alzheimer’s disease (Gu et al., 2014), the large leaching of Zr may cause cytotoxicity (Li et al., 2012), and how rare earth elements are metabolized in the human body is not clear, which limits the application of these elements (Staiger et al., 2006). Therefore, it is imperative to develop biomedical Mg alloys with biosafe elements. As common elements in the human body, both Ca and Zn elements possess good biocompatibility (Chang et al., 2020). The corrosion resistance properties and the *in vivo* degradation behavior of Mg-Zn and Mg-Ca alloys have been well studied in recent years (Jafari et al., 2017; Holweg et al., 2020; Schäublin et al., 2022). However, the second phase of binary alloys is difficult to modulate from the perspective of second phase micro electric corrosion. It has been shown that the second phase in Mg-Ca alloys is dominated by the Mg_2_Ca phase, which potential is about −50 mV relative to the Mg matrix, and exhibits preferential corrosion over the matrix (Bahmani et al., 2019; Moreno et al., 2019). The second phase of Mg-Zn alloy is dominated by the MgZn phase, which has an electric potential of about 550 mV higher than that of the Mg matrix and may cause severe micro electric corrosion, and weaken the corrosion resistance (Jin et al., 2020).

For the ternary Mg-Zn-Ca alloys, different contents of Zn and Ca lead to variations in the nature of the second phase (Zhang et al., 2011; Bakhsheshi-Rad et al., 2012). When the Zn/Ca mass ratio is greater than 5, the MgZn + Ca_2_Mg_6_Zn_3_ phases tend to form in Mg-Zn-Ca alloy. When the mass ratio is between 5 and 2, Ca_2_Mg_6_Zn_3_ phase is easily generated. With further increase Ca content, the Mg_2_Ca phase starts to precipitate out from the alloy matrix, and the main second phases are mainly Mg_2_Ca + Ca_2_Mg_6_Zn_3_ phase when the Zn/Ca mass ratio is below 2. For the above three types of second phases, the potential of Ca_2_Mg_6_Zn_3_ phase is about 80 mV higher than that of the matrix (Fu et al., 2022), which has the smallest potential difference among these second phases. Therefore, avoiding the formation of MgZn phase in Mg-Zn-Ca alloy and increasing the proportion of Ca_2_Mg_6_Zn_3_ phase will help to improve the corrosion resistance of Mg-Zn-Ca alloy.

From the perspective of ensuring the plasticity of Mg alloy, the content of Ca element in Mg alloy needs to be controlled below 0.3 wt. % (Zhang et al., 2012; Schäublin et al., 2022). In this case, reducing Zn content helps to generate the Ca_2_Mg_6_Zn_3_ phase in Mg-Zn-Ca alloy. However, reducing the Zn and Ca content simultaneously will lead to a decrease in mechanical properties of Mg alloys. Consequently, the inclusion of reinforcing phases is necessary to compensate the mechanical properties due to the low content of Zn and Ca. The conventional reinforce phases such as graphene oxide (GO) (Shuai et al., 2019), SiC (Ali et al., 2019) and TiC (Nie et al., 2020) are not suitable to add in biomedical Mg alloy due to their non-degradable property. In contrast, MgO particles are degradable and they can maintain excellent interfacial bonding with the Mg matrix (Lin et al., 2018; Fan et al., 2022). Besides, MgO can simultaneously impede grain growth by pinning grain boundaries (Fan et al., 2022) and enhance the mechanical properties through Orowan strengthening (Goh et al., 2007). Moreover, the degradation product of MgO was the same with the Mg matrix (Mg(OH)_2_), which could enhance the densities of the corrosion product layer (Tang et al., 2023).

On this basis, the influence of the second phase on the corrosion resistance of the Mg-Zn-Ca alloy was further investigated in this work. Extruded low alloyed 1.0MgO/Mg-1Zn-0.2Ca (ZX) composite was the studied alloy and 1.0MgO/Mg-3Zn-0.2Ca (3ZX) was regarded as a comparison to study the effect of the second phase on the *in vitro* corrosion performance and to evaluate the *in vivo* corrosion behavior of ZX.

## 2 Materials and method

### 2.1 Materials preparation

**FIGURE 1:**
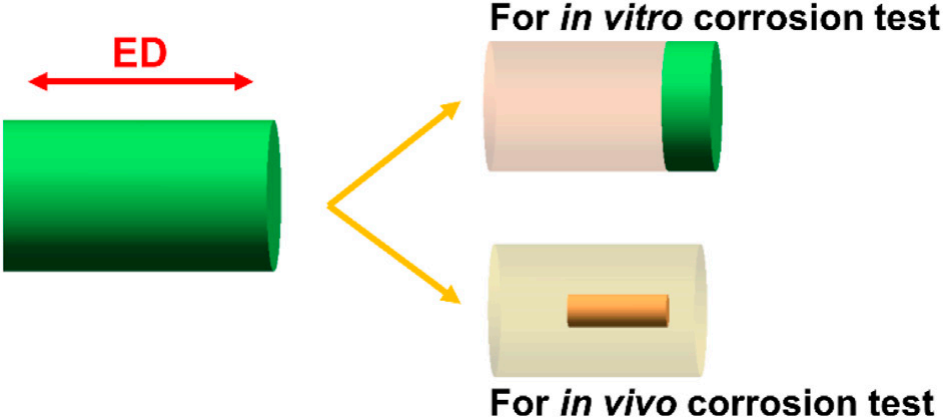
The schematic diagram of the samples cut from the extruded bars.

The as-cast ZX and 3ZX composites used in the current study were prepared by combining pure Mg (wt% 99.99%), pure Zn (wt% 99.99%) and Mg-25Ca (wt%) master alloys at a temperature of 780°C in the presence of an inert gas atmosphere consisting of N_2_ and SF_6_. MgO nanoparticles (MgO NPs) were preheated at 200°C in a muffle furnace and then were introduced into the melt at 780°C using a high shear stirrer and ultrasonic treatment (Fan et al., 2011). The mixture melt was poured into a cylindrical ingot with a diameter of 60 mm. After the homogenization treatment (400°C/24 h for 3ZX and 420°C/8 h for ZX), the extrusion process was carried out at 300°C with an extrusion ratio of 56 and the rod with a diameter of 8 mm was obtained. All the test specimens were then obtained by cutting the extruded rods (the schematic diagram shown in Figure 1).

### 2.2 Microstructure characterization

The phase types were determined by X-ray diffraction (XRD, D/MAX-2500, Japan, Cu-Kα, 20 - 80) with a scanning speed of 5/min and an acceleration voltage of 40 kV and current of 40 mA. The specimens for scanning electron microscope (SEM) observation were prepared by mechanical grinding and polishing followed by etching with picric acid solution (picric acid 2.75 g, acetic acid 2.5 mL, ultrapure water 5 mL, anhydrous ethanol 45 mL) and the average grain size was determined by ImageJ software. The energy dispersive spectrometer (EDS) was used to analyze the second phase composition. The detailed microstructure was analyzed by transmission electron microscopy (TEM). TEM samples were prepared as standard 3 mm thick cylinders by mechanical grinding to a thickness of approximately 50 μm, followed by Ar + ion milling to achieve electron transparency. Imaging was performed in scanning-TEM (STEM) mode using a high-angle annular dark field (HAADF) detector and the chemical information was obtained in STEM mode using an EDS detector.

### 2.3 Electrochemical test

The electrochemical polarization and electrochemical impedance spectroscopy were conducted in simulated body fluid using a Zennium electrochemical workstation with a three-electrode test system. The simulated body fluid was prepared with NaCl (6.5453 g), NaHCO3 (2.2683g), KCl (0.3728 g), NaHPO4·7H2O (0.2681 g), MgCl2·6H2O (0.3050 g), CaCl2 (0.2780 g), Na2SO4 (0.0711 g) and (CH2OH)5CNH2 (6.057 g). The reference electrode used was a saturated glycerol electrode, and the auxiliary electrode was a graphite electrode. The sample used for the electrochemical tests was cut into a cylinder shape with a diameter of 8 mm and a height of 3 mm. A 60-min open-cycle potential (OCP) test was carried out first to ensure that the sample potential was stable for subsequent tests, and the scanning speed was 1 mV/s. Potentiodynamic polarization experiments were performed within a range of −500 mV–500 mV. During the electrochemical impedance spectrum (EIS), the test frequency ranged from 0.01 Hz to 100 kHz, and the amplitude of the positive spin perturbation signal was 5 mV. To ensure accuracy, three parallel samples were tested in each group.

### 2.4 *In vitro* degradation

The *in vitro* degradation rate of 1ZX1.0 and 3ZX1.0 were evaluated by weight loss measured by immersion test in SBF solution. Three parallel samples with a surface area of 176 mm^2^ were prepared by mechanically grinding the surface with SiC5000 paper. The SBF solution was added at a rate of 0.25 mL/mm^2^. Three parallel samples were set up in each group for immersion test in water bath. All the samples are pre-weight (*m*_0_) before immersion. Change the corrosion medium every 48 h and record the pH value of each group. After immersion for different days, the samples were picked and cleaned by chromic acid solution (CrO_3_ 180 g/L, AgNO_3_ 10 g/L, BaNO_3_ 10 g/L) for 2–3 min to remove surface corrosion products. After that, the weight of the cleaned sample was measured as *m*_1_. The annual corrosion rate of the samples was then calculated according to the following Eq. 1.

$$C=\frac{K\times \left(m_{0}-m_{1}\right)}{\rho \times A\times t}$$ (1)

The cross section of the immersed sample was measured by SEM. The sample was first set vertically into the resin and then cut open using a diamond cutter to reveal the cross section. After that it was prepared by mechanical grinding and polishing and the element contents in different positions were measured by EDS.

### 2.5 *In vivo* degradation experiment

**FIGURE 2:**
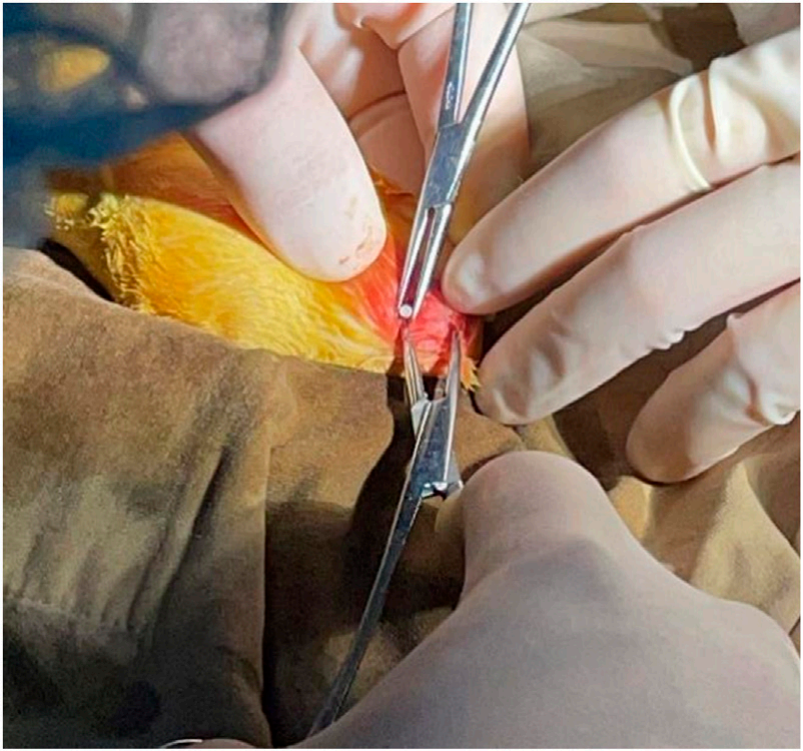
Implantation process of ZX composite.

Degradation experiments were carried out using with better. The presented material was implanted in adult Japanese white rabbits (provided by *Tianjin Institute of Medical Sciences*) to examine its corrosion resistance *in vivo*. The extrusion rods with a diameter of 8 mm were machined into cylindrical implants with a diameter of 2 mm and a length of 6 mm, which were sterilized by Co60 radiation before surgery. During the operation, the rabbit was anesthetized and the fur on the hind limbs was removed. A hole defect was made in the left femoral condyle with a diameter of about 2 mm. The implant was inserted into the hole and the sutured the myofascial membrane and skin after operation. The operation process was shown in Figure 2. The rabbits were euthanized at 6- and 13-weeks post operation. The Femoral tissues (including implant) were then fixed in 4% paraformaldehyde solution.

The micro-CT scanning was then carried out by Inveon MM CT (SIEMENS, Munich, Germany). The Inveon Acquisition Workplace (SIEMENS, Munich, Germany) and Inveon Research Workplace (SIEMENS, Munich, Germany) software were then used for scanning and data analysis. The COBRA_Exxim (EXXIM Computing Corp., Livermore, CA) software was used for 3D reconstruction and bone parameter analysis.

### 2.6 Potential measurement

Scanning kelvin probe force microscopy (SKPFM) is used to measure the relative potential between second phase and matrix. The sample used in experiment was cylindrical with diameter of 8 mm and height of 3 mm. It was grinded and then was polished mechanically before examination.

## 3 Results

**FIGURE 3:**
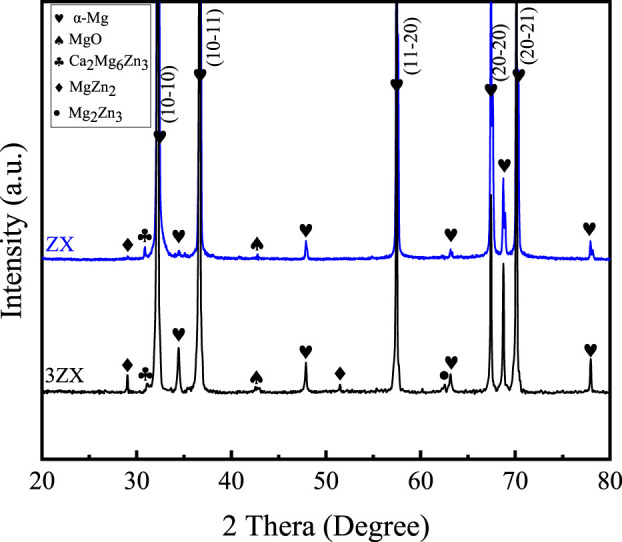
The XRD spectra of as-extruded ZX and the 3ZX composites.

Figure 3 displays the XRD spectra obtained perpendicular to the extrusion direction (ED) of the as-extruded ZX and 3ZX composites. The obtained results indicate that the main diffraction peaks of both materials predominantly align with the prismatic plane and pyramidal plane. This indicates that both materials show a typical basal texture (Jiang et al., 2018). Additionally, a slight distinction is observed in the diffraction pattern of the second phase between the two materials. In the spectrum of the 3ZX composite, the peaks corresponding to MgZn_2_, Mg_2_Zn_3_ are evident and a few peaks of Ca_2_Mg_6_Zn_3_ also appeared, indicating that the second phase in the 3ZX composite is mainly MgZn phase accompanied by a small amount of Ca_2_Mg_6_Zn_3_ phase. In addition, the XRD spectrum shows a small diffraction peak corresponding to MgO. However, as the Zn content reduced to 1.0 wt% in ZX composite, most of the diffraction peaks of the MgZn phase disappear, indicating a change of second phase from the existence of Mg-Zn and Ca_2_Mg_6_Zn_3_ phases in 3ZX to the dominated Ca_2_Mg_6_Zn_3_ phase in ZX.

**FIGURE 4:**
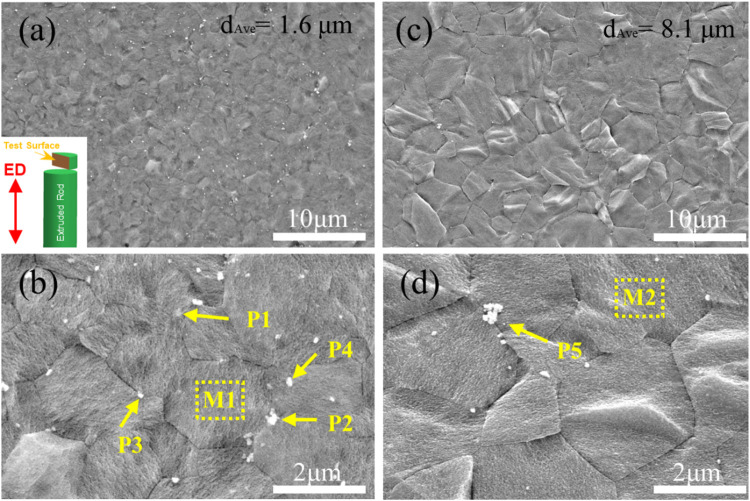
SEM microstructure of as-extruded **(A, B)** 3ZX and **(C, D)** ZX composites.

Figure 4 exhibits the SEM images of as-extruded ZX and 3ZX composites taken along the ED at various magnifications. The low magnification images (Figures 4A, C) reveal that the ZX composite has an average grain size of approximately 8.1 μm, whereas the 3ZX composite exhibits an average grain size of around 1.6 μm. The discrepancy in grain size is attributed to the Zn content and the second phase. As shown in Table 1, the Zn content in ZX composite is reduced to 0.41 at. % from 1.01 at. % in 3ZX, and this large reduction weakens the grain refinement capability. Furthermore, the reduced volume fraction and size of the second phase also decrease the pinning effect of grain boundary migration during recrystallization. The high magnification images (Figures 4B, D) show numerous white particles are distributed uniformly in the 3ZX, and the volume fraction of them is about 2.69%. The EDS results in Table 1 and the phase composition in Figure 3 classify these secondary phases as MgO nanoparticles labeled P1 and P2, Mg-Zn phases labeled P3, and Ca_2_Mg_6_Zn_3_ phases labeled P4 in 3ZX. While for ZX, the fraction of the second phase in is reduced to 1.86%, and only a few Ca_2_Mg_6_Zn_3_ phases are observed at the grain boundaries. Moreover, the mean diameter of the second phase is also decreased. The high O element content of MgO NPs confirmed by EDS results (Table 1) at the intersection of grain boundary in ZX is also observed and labeled P5 in Figure 4D.

**TABLE 1:** EDS results of ZX and 3ZX in Figure 4.

Analysed locations	Alloys	Elements (at. %)			
		Mg	Zn	Ca	O
M1	3ZX	98.89	1.01	0.10	-
P1	3ZX	73.64	1.22	0.02	25.30
P2	3ZX	50.18	1.30	0	48.52
P3	3ZX	96.46	3.54	0	-
P4	3ZX	98.93	1.35	0.26	-
M2	ZX	99.52	0.44	0.04	-
P5	ZX	72.03	0.41	0.10	27.46

**FIGURE 5:**
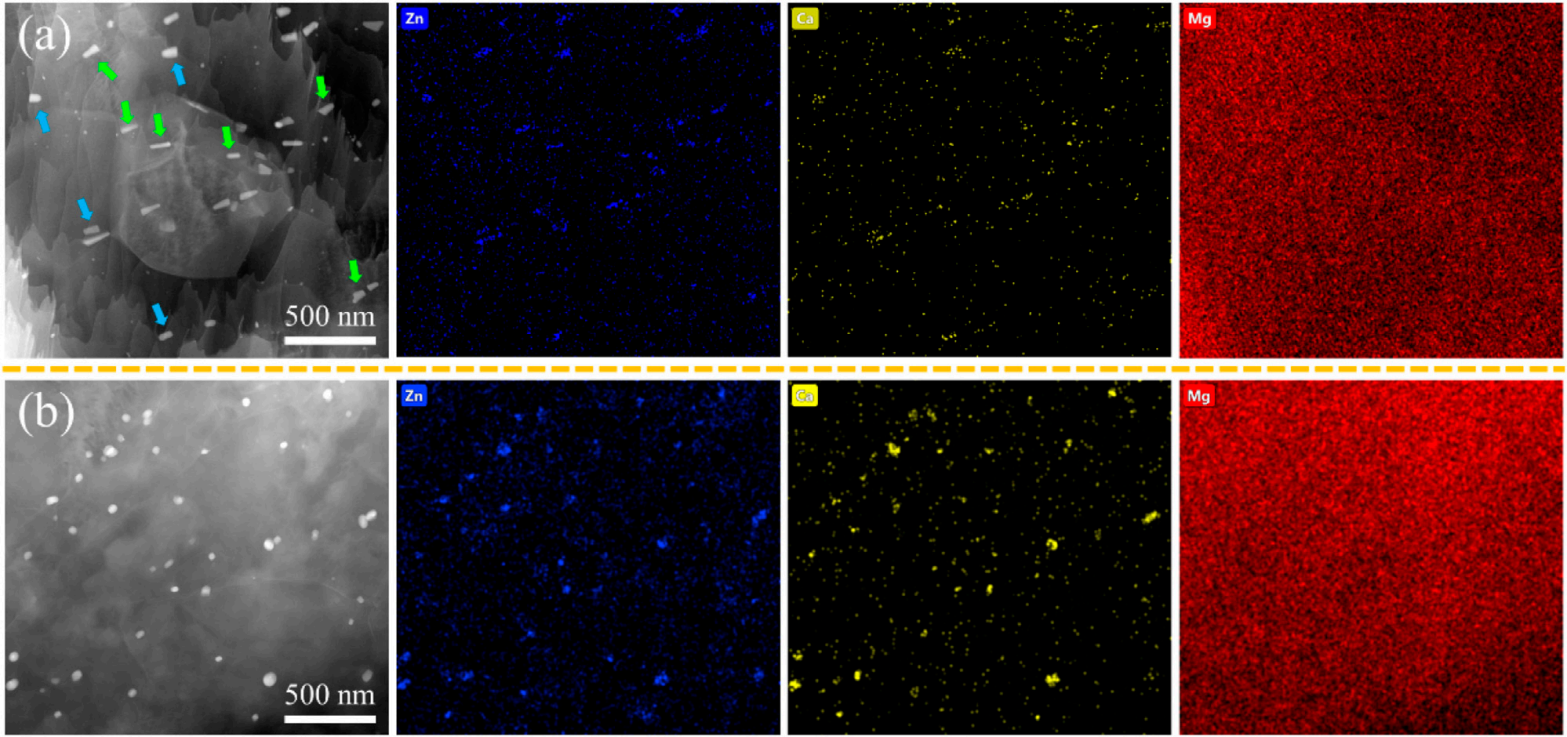
HAADF images of as-extruded **(A)** 3ZX and **(B)** ZX composites.

Figure 5 depicts the TEM results of as-extruded ZX and 3ZX composites. From the HAADF image and EDS mappings of 3ZX in Figure 5A, the dark and elongated second phases marked by green arrows are the MgZn phases, and the bright blocky second phase marked by blue arrows, which contains both Ca and Zn elements, is the Ca_2_Mg_6_Zn_3_ phase. This finding further corroborates the domination of the MgZn phase in 3ZX. In contrast, most of the second phase in ZX is predicted to be Ca_2_Mg_6_Zn_3_ phase and its volume fraction is also decreased. The mean diameter of the second phase in ZX is 60 nm, which is 50% smaller than the 127 nm in 3ZX.

**FIGURE 6:**
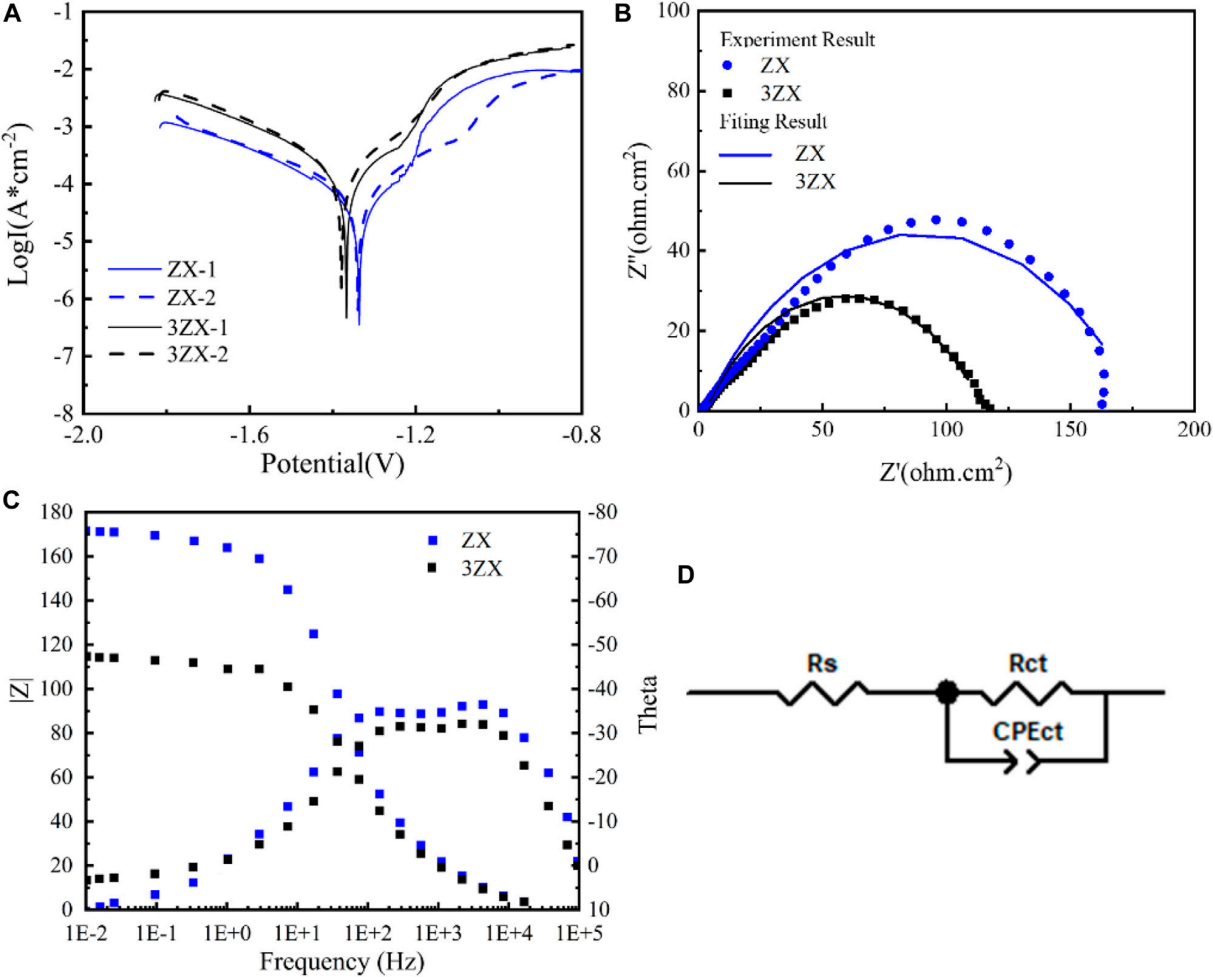
Electrochemical test result of as extruded ZX and 3ZX **(A)** Potential dynamic polarization curves, **(B)** Nyquist spectra, **(C)** Bode plots, **(D)** Corresponding equivalent circuits.

Figure 6 presents the results of the electrochemical tests conducted on the as-extruded ZX and 3ZX composites. Figure 6A displays the polarization curve from twice measurements for each composite. Typically, the current density in the cathodic curve represents the hydrogen evolution rate due to the reaction of the Mg matrix with the caustic medium, whereas the anodic current density is attributed to the dissociation rate of the Mg matrix into Mg^2+^.The current density of the ZX composite, both cathodically and anodically, is significantly lower as compared to the 3ZX composite, indicating less matrix dissolution activation and a lower hydrogen release rate. Moreover, the *E*_*corr*_ of the ZX has improved to −1.367 V, and the *I*_*corr*_ has reduced to 27.5 μA as shown in Table 2, which indicates a higher corrosion resistance in the matrix dissolution reaction than that of 3ZX. Additionally, the passivation potential of the ZX is lower than that of the 3ZX, which enhances its capability to form a passivated film. Figure 6B illustrates the electrochemical impedance spectrum (EIS), where the scatter plot reflects the experimental results and the curve plot portrays the fitted results. Figure 6C presents the Bode plot; Figure 6D shows the equivalent circuit fitted using Zviwe software. In the circuit, *R*_*s*_ indicates the solution resistance between the working electrode and reference electrode, the *CPE*_*ct*_ *-C* represents the charge of the bilayer equivalent capacitance, and *R*_*ct*_ denotes the charge transfer resistance. Both 3ZX and ZX composites exhibit only a large capacitive loop associated with the *R*_*ct*,_ which corresponds to the phase peak in the high frequency range within the Bode plots. However, the ZX composite exhibits a larger radius of the capacitive loop, with *R*_*ct*_ increasing from 108 Ω in 3ZX to 170 Ω in ZX. This implies a low matrix dissolution activity of ZX, resulting in an augments of the material’s corrosion resistance. The average corrosion rate of ZX, as estimated by the electrochemical tests, is 0.73 mm/y, which is lower than that of the 3ZX composite (1.06 mm/y).

**TABLE 2:** Corrosion properties of 3ZX and ZX composites.

Samples	*E*_corr_ (V)	*I*_corr_ (μA/cm^2^)	*Rs* (Ω)	*CPEct-C* (E)	*Rct* (Ω)	*CR*(mm/y)
3ZX	−1.336	86.5 ± 2	7.48	4.08e-5 (0.0000508)	108	1.06
ZX	−1.367	27.5 ± 5	7.28	3.62e-5 (0.0000362)	170	0.73

**FIGURE 7:**
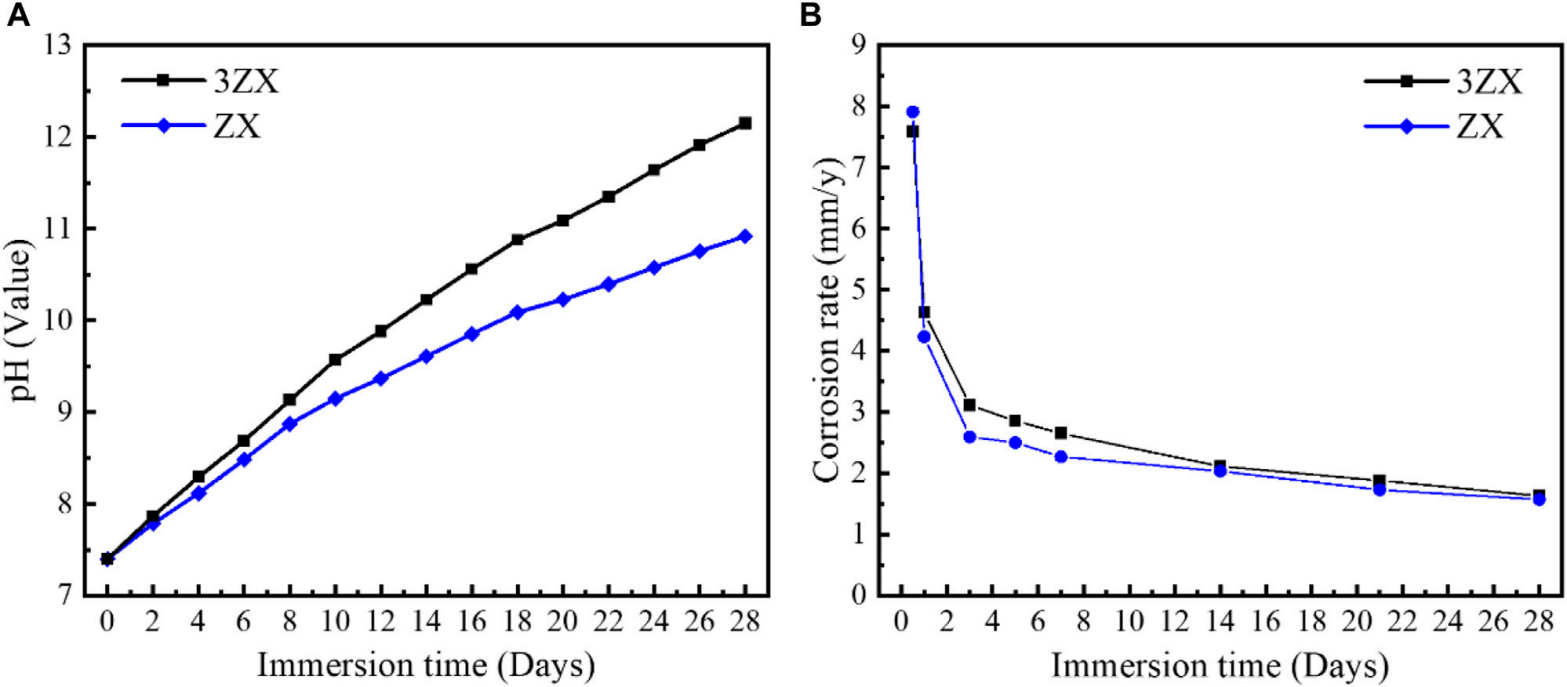
**(A)**Accumulated pH change curve, **(B)** average corrosion rate.

Figure 7 displays the accumulated pH value curves of the as-extruded ZX and 3ZX composites during *in vitro* immersion experiment (28 days) and the average corrosion rate calculated by weight loss method. The results show that the pH variation curve of the 3ZX composite maintains a stabilized high slope during the entire immersion test, resulting in a higher accumulated pH value as compared to the ZX. This indicates a high corrosion rate and a poor ability to form protective corrosion products layers. Conversely, the slope of the accumulated pH change curve of the ZX composite decreases significantly after the 8th day post-immersion and remains at a lowslope until the end of the immersion. This low growth rate of pH value in the ZX composite also indicates a low average corrosion rate and a delayed dissociation rate of the Mg matrix. The changes in the average corrosion rate of the ZX and 3ZX composites, as shown in Figure 7B, are consistent with the pH variations. The 3ZX composite maintains a high corrosion rate during the immersion test, and the average corrosion rate at the 28th day post-immersion is 1.63 mm/y, while for ZX is 1.57 mm/y.

**FIGURE 8:**
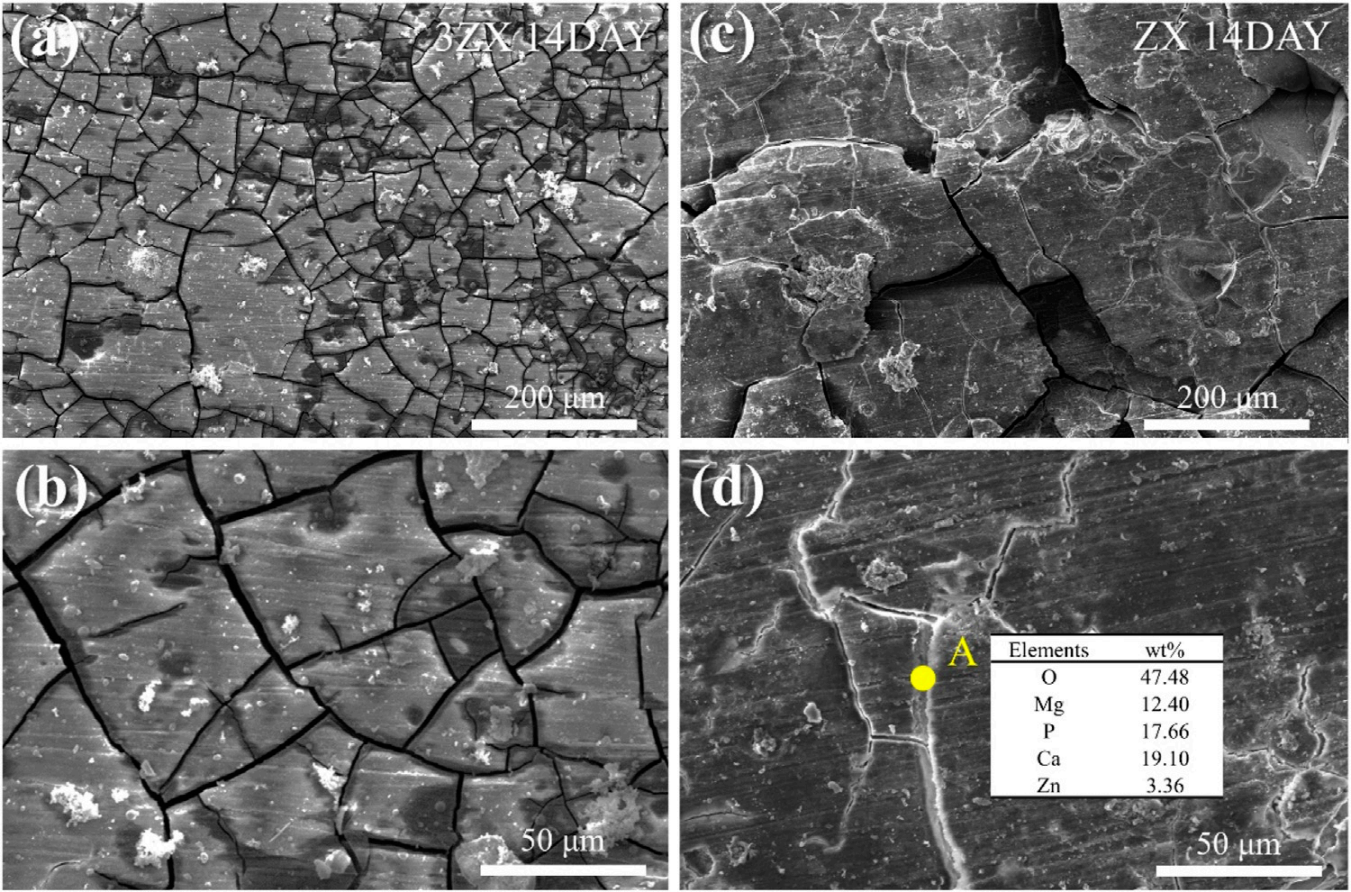
Surface morphology after immersion for 14 days: **(A, B)** 3ZX and **(C, D)** high ZX.

**FIGURE 9:**
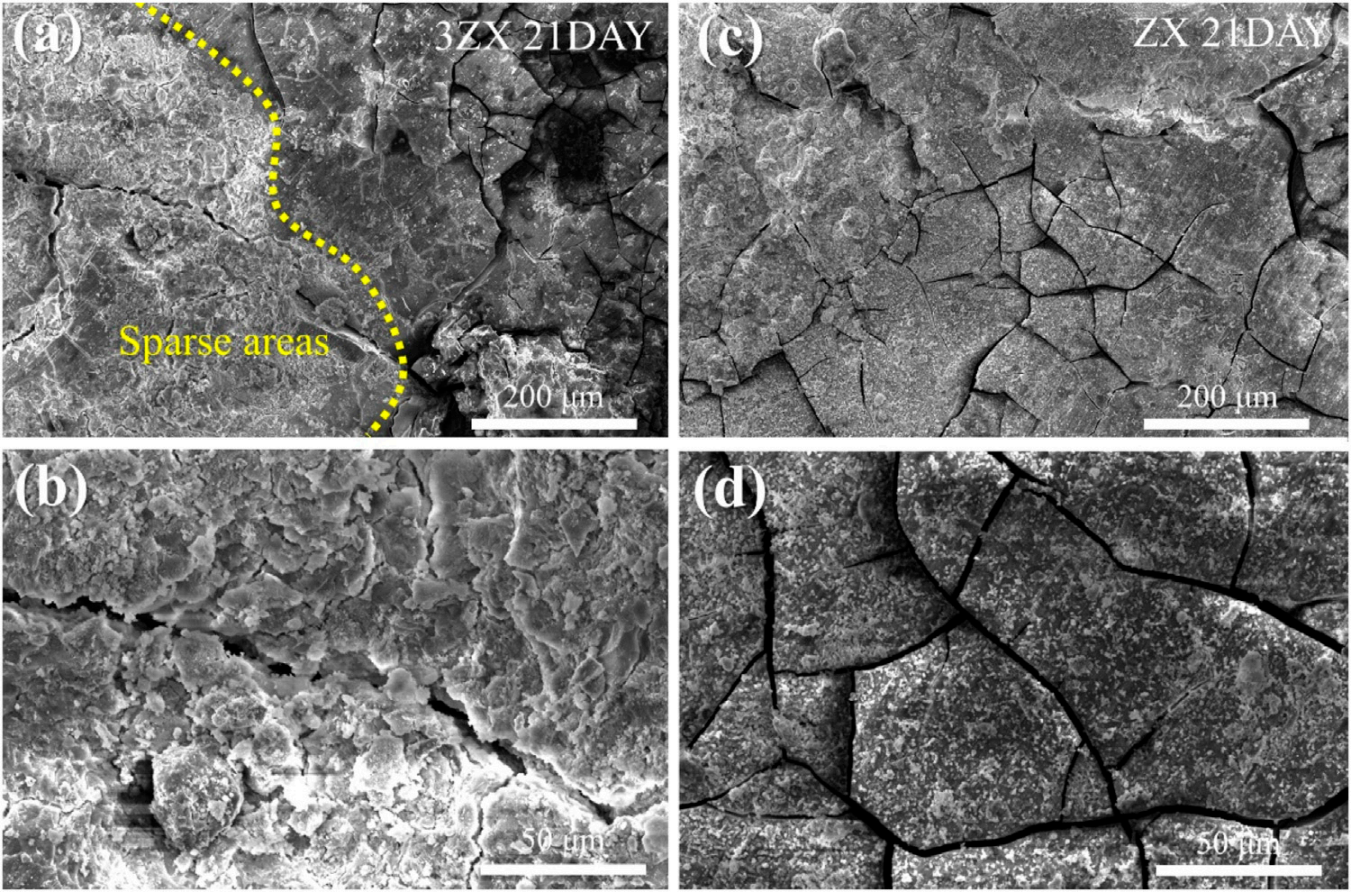
Surface morphology after immersion for 21 days: **(A, B)** 3ZX and **(C, D)** ZX.

Figures 8, 9 present the surface morphologies of the as-extruded ZX and 3ZX composites at 14-day and 21-day post-immersion in SBF solution, respectively. The results demonstrate that both ZX and 3ZX composites exhibit a smooth surface appearance after 14 days of immersion. The corrosion product layer completely covers the surface of both specimens, and minimal pitting corrosion is observed. However, severe cracking is observed on the 3ZX composite. Some deep cracks are formed in the corrosion products layer in 3ZX composite, resulting in a black mesh distribution under the electron microscope. This leads to a division of the corrosion product layer into smaller areas and reduces its protective effect. On the contrary, the proportion of deep cracks in the corrosion product layer of the ZX composite is significantly lower, allowing the corrosion product layer to provide a large block covering on the material’s surface. Nonetheless, shallow cracks are still visible on these blocks. Based on the EDS results in Figure 8D, the element contents in the shallow cracks on the surface of ZX are still mainly composed of Ca and P.

After extending the immersion time to 21 days, the density of deep cracks on the surface of the corrosion product layer of the 3ZX composite further increases. The corrosion product layer becomes divided into fine areas, making it easier for the corrosive medium to penetrate the inner layer. Additionally, part of the corrosion product layer on the 3ZX composite changes from dense to sparse, as shown in the bright white area on the left side of Figure 9A. The sparse area displayed in Figure 9B is distinct from the dense Ca-P particle deposition area (Figure 9D), and the corrosion product layer starts to flake off, significantly decreasing the protective effect of the corrosion product layer on the matrix. Conversely, the ZX composite can maintain a dense corrosion product layer, and minimal sparse areas are observed. These findings suggest that the corrosion product layer on the ZX composite is denser and more complete, making it more effective at shielding the matrix from corrosion.

**FIGURE 10:**
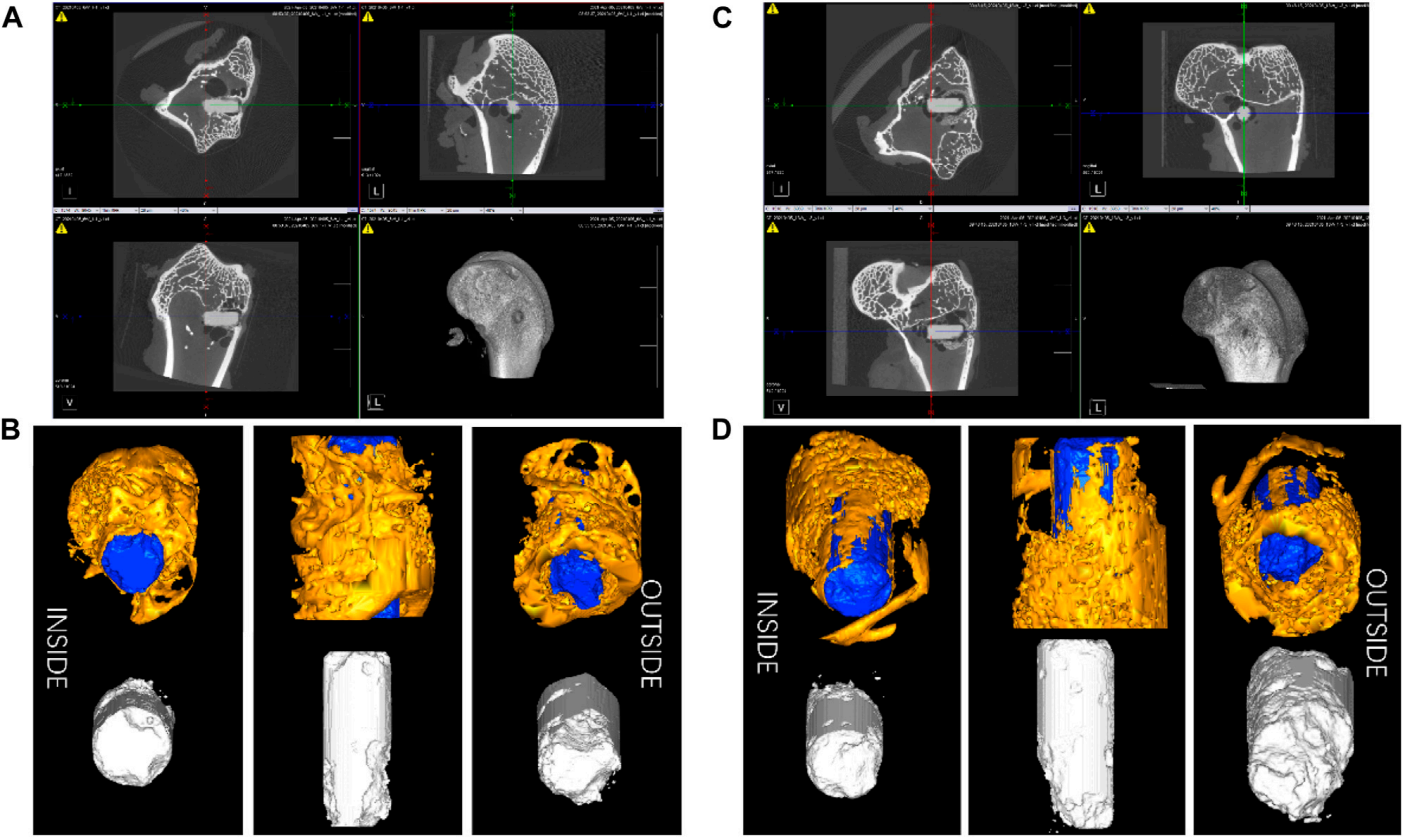
The 2-D cross-sectional pictures of post-surgery femoral ankle of the thigh and implants **(A)** 6weeks and **(C)** 13 weeks, and the 3-D reconstruction image of the implants and bone tissue within a 1 mm radius surrounding the implants **(B)** 6weeks and **(D)** 13 weeks.

The *in vivo* degradation experiments were conducted with the ZX material due to its better corrosion resistance. Figure 10 displays 2-D cross-sectional images of femoral ankles and implants after surgery, along with a 3-D reconstruction of the implants and surrounding bone tissue within a 1 mm radius. The images demonstrate that the degradation rate outside the implant is notably higher than that inside the implant. This is possibly attributed to the presence of blood vessels in the fascia surrounding the femur. The flow of blood causes an increased rate of ion exchange and degradation outside the implant. The mean starting volume of the implant rods was 15.9 ± 0.4 mm^3^. After 6 weeks post-operation, an initial implant degradation of 12.97%. After 13 weeks post-operation, the original implant volume had degras by approximately 23.31% to 12.2 ± 0.3 mm^3^. The calculated degradation rate of ZX material in weeks 1-6 is around 0.75 mm/y; the average degradation rate in weeks 6–13 is 0.60 mm/y. The decreased degradation rate contributed to an improvement in the bone density (BMD) around the implant, as evidenced by increased trabecular number (Tb. N.) and decreased trabecular separation (Tb. Sp.), as shown in Table 3. These changes indicate that the bone is growing well around the implant.

**TABLE 3:** Information on the bone tissue surrounding the implant.

Times	Degradation Rate (%)	BV/TV	BMD (mg/cm^3^)	Tb.Th (mm)	Tb.N (1/mm)	Tb.Sp (mm)
6 Weeks	12.97	20.29	1173.25	0.11	1.79	0.44
13 Weeks	23.31	28.09	1371.29	0.13	2.06	0.34

## 4 Discussion

The objective of this study is to determine whether MgO/Mg-Zn-Ca composites with low Zn/Ca ratio has a greater corrosion resistance than that with higher ratios. Generally, refining grain size increases the density of the corrosion product layer (Aung and Zhou, 2010; Luo et al., 2020), which effectively separates the alloy matrix from the corrosion medium, and enhances the material’s corrosion resistance (Tang et al., 2023). However, the corrosion resistance of the ZX composites with larger grain size is superior to that of the 3ZX composites with smaller grain size from both electrochemical tests and immersion experiments. Electrochemical tests reveal that ZX composite has lower corrosion currents in both cathodic and anodic regions of the polarization curve, indicating that they exhibit lower electrochemical corrosion activity (Abdel-Gawad and Shoeib, 2019). The passivation phenomenon of ZX is more noticeable in the anodic region of the polarization curve. This indicates that the surface of ZX is more prone to form a passivation film. The EIS results that the passivation film on the surface of ZX exhibits a large capacitative loop, which significantly reduces the transfer of current between the matrix and corrosion medium during the corrosion process and ultimately protects the matrix from corrosion. Although electrochemical tests can only explain the short-term corrosion behavior, the above conclusions could illustrate that the key to the higher corrosion resistance of ZX composite is the formation of a strong protective corrosion product layer during the corrosion process.

**FIGURE 11:**
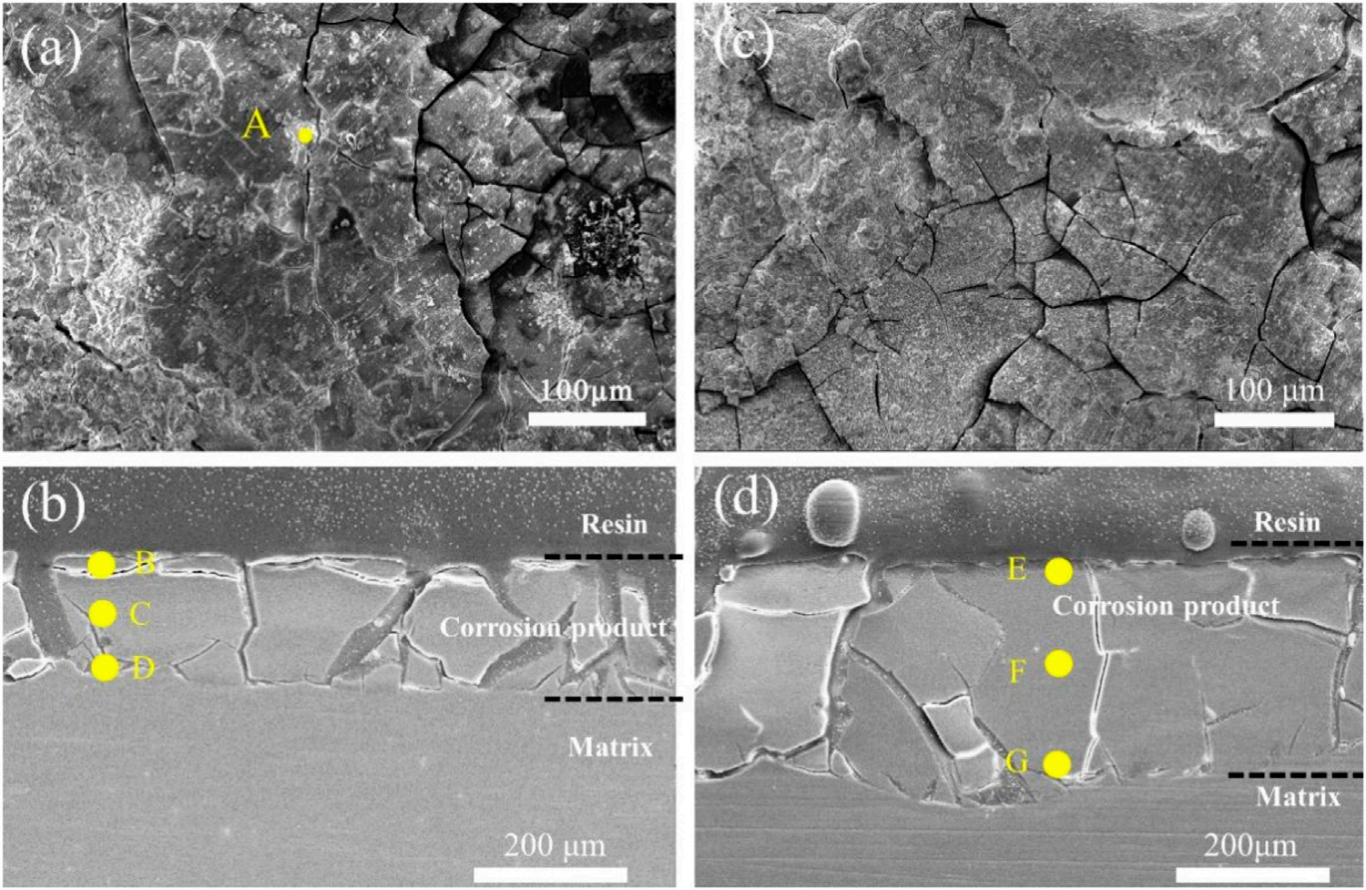
**(A)** Surface image of 3ZX composite after 21 days of immersion, **(B)** cross-section image of 3ZX composite after 21 days immersion; **(C)** surface image of ZX composite after 21 days immersion, **(D)** cross-section image of ZX composite after 21 days immersion.

Figure 11 displays the surface and cross-sectional images of 3ZX and ZX composites after 21 days of immersion, with the EDS results tabulated in Table 4. EDS results showed that the surface cracks of 3ZX composites were covered with high Zn content at point A. Cross-sectional images of Figures 11B, D, and the corresponding EDS results, reflected similar results. The corrosion product layers of 3ZX composites were divided into two layers. The top layer at point B had a Ca-P product layer rich in Zn elements. The layer beneath B, at points C and D, contained a Mg(OH)_2_ layer with minimal Ca and P elements. Point C was roughly at the interface between the external Ca-P layer and the internal Mg(OH)_2_ layer and had relatively high Ca and P content. Point D, located close to the matrix, had the least content of Ca and P elements in Mg(OH)_2_ layer. Moreover, the Mg-Zn phase released during the degradation of the surrounding matrix remained in the corrosion product layer leading to higher Zn content at point D than in the matrix (Table 1, M1). In contrast, the cross-section of ZX composite was distinctly different from that in 3ZX. EDS results at the E point on the surface of corrosion product layer, and at the F and G points within the corrosion product, revealed no obvious Zn enrichment in the corrosion product layer of ZX due to the reduction of the Mg-Zn phase in ZX. Comparing the element content of points E, F and G, it is evident that E and F points contain higher Ca and P elements, which are closer to the element content of the Ca-P layer, indicating a higher thickness of the corrosion product layer deposited on the ZX. Moreover, by comparing the cross-sectional photos of ZX and 3ZX, it is evident that there are fewer cracks in the corrosion product layer on the surface of ZX.

**TABLE 4:** EDS results of 3ZX and ZX in Figure 11.

Analysed locations	Alloys	At. (wt%)				
		Mg	Zn	Ca	P	O
A	3ZX	10.07	5.10	7.51	11.34	65.97
B	3ZX	8.36	3.63	15.42	17.65	54.59
C	3ZX	18.29	0.80	5.46	7.47	67.69
D	3ZX	22.28	1.12	3.13	1.93	71.53
E	ZX	9.70	1.09	7.15	9.34	72.72
F	ZX	17.42	0.96	7.74	10.27	63.62
G	ZX	24.82	0.92	3.14	1.72	69.40

**FIGURE 12:**
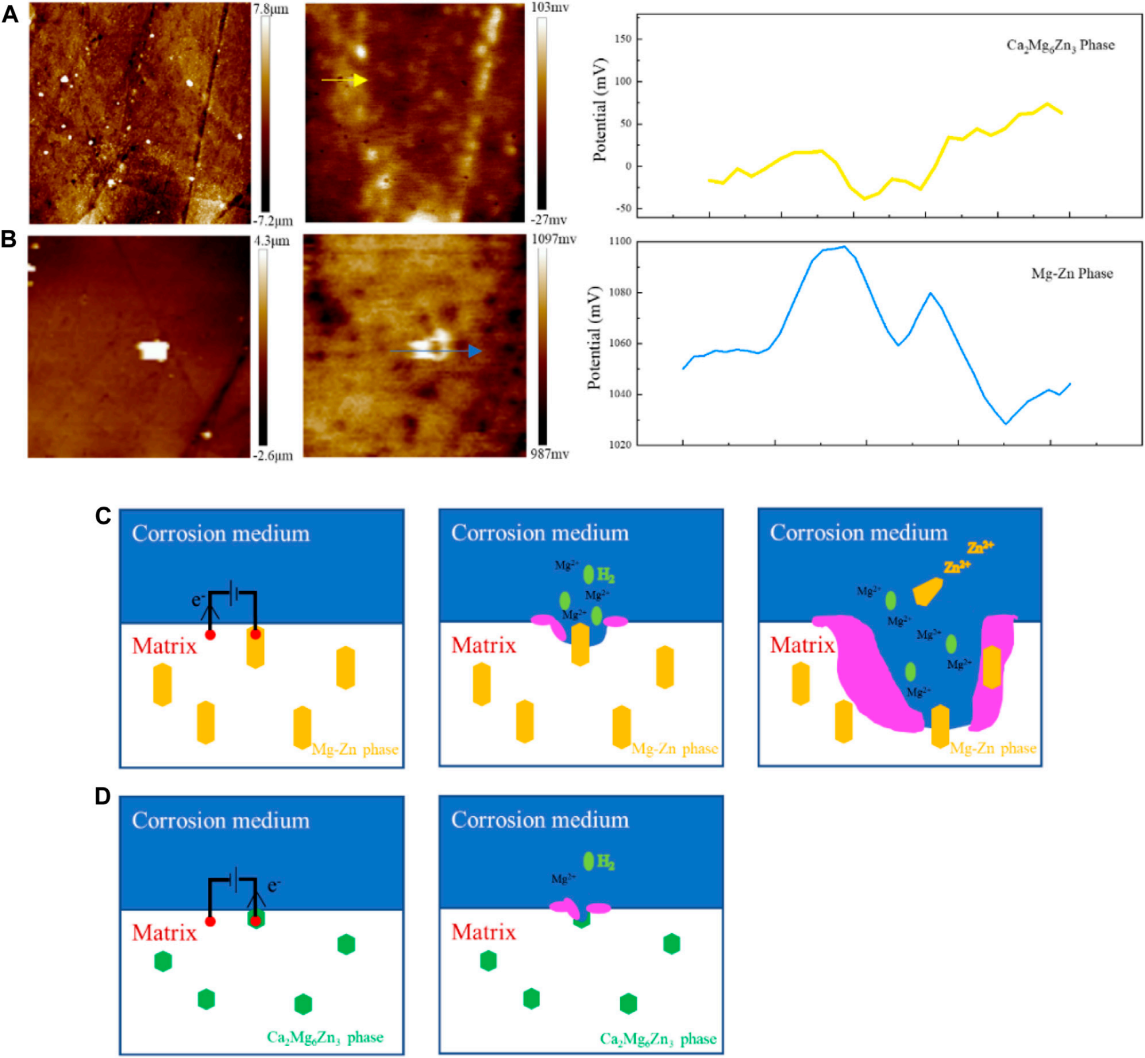
Surface height distribution and potential distribution of **(A, B)** 3ZX measured by SKPFM, **(C)** schematic diagram of 3ZX and **(D)** ZX corrosion process.

The changes in the composition of the second phase in ZX composites lead to significant changes in corrosion behavior. Earlier studies have shown that the potential difference between the Mg-Zn phase and the Mg matrix is nearly ten times that of the Ca_2_Mg_6_Zn_3_ phase (Jin et al., 2020). The SKPFM test results presented in this study (Figures 12A, B) also support similar conclusions. The study shows that although the Mg-Zn phase does not show significant potential difference in 3ZX as it has been reported due to matrix material changes, the potential difference of Mg-Zn phase is still about 60 mV higher than that of the matrix. The potential of Ca_2_Mg_6_Zn_3_ phase is about 40 mV lower than that of the matrix. Thus, the presence of the Mg-Zn phase in the 3ZX composite can cause severe galvanic corrosion. The matrix of lower electrode potential acts as the anode in the electrogalvanic corrosion process and is preferentially corroded, while the Mg-Zn phase acts as the cathode and does not degrade due to the protection of anode sacrifice. As illustrated in the schematic diagram of the corrosion process in Figure 12C, when the Mg matrix surrounding the Mg-Zn phases dissolves, the Mg-Zn phase without clutching to the matrix falls off from the surface of the matrix. Part of these fallen Mg-Zn phases may enter the corrosion medium and slowly degrade with time. The other part may remain on the surface of the sample with the gradual deposition of Ca-P products and eventually form Zn-containing corrosion products. Under the influence of galvanic corrosion, the corrosion rate and hydrogen release rate of 3ZX are accelerated, resulting in internal destruction of the corrosion product layer and more cracks in the corrosion product layer of 3ZX composite.

In ZX composite, the decreased potential difference between matrix and second phases, which ultimately reduces the micro-galvanic corrosion and improves the uniformity of the corrosion product layer. The Ca_2_Mg_6_Zn_3_ phase in ZX acts as an anode during electrochemical corrosion, as shown in Figure 12D, due to its lower potential than the matrix. Additionally, the reduced potential difference between matrix and second phase in ZX helps to prevent severe local corrosion and improve the density of the corrosion product layer (Tie et al., 2022). As a result, the corrosion rate of Mg matrix reduces under the protection of Ca_2_Mg_6_Zn_3_ phase. Furthermore, the presence of Ca_2_Mg_6_Zn_3_ phase significantly weakened the micro-galvanic corrosion, resulting in a reduction in the fraction and width of cracks in the corrosion product layer. The surface of corrosion product layer of ZX is more complete than that of 3ZX composite. The deep cracks on the surface of ZX composite practically disappear, whereas some shallow cracks remain. The EDS results inside the shallow cracks (Figure 9D) reveal that the element content inside and on the surface of the corrosion product layer is similar. Accordingly, these shallow cracks only form on the surface of the corrosion product layer and do not affect the layer’s protective performance. This implies that the corrosion product layer of ZX composite effectively protects the matrix and reduces the annual corrosion rate to 1.57 mm/y.

## 5 Conclusion

In this study, we analyze the distinction in the corrosion behavior of 3ZX and ZX composites after the reduction of Zn/Ca ratio. The main conclusions are as follows:

The reduced content of Zn in ZX composites alters the second phase from the cathode Mg-Zn phase, which has a larger potential difference, to the anode Ca_2_Mg_6_Zn_3_ phase, which has a smaller potential difference. This alteration effectively prevents severe local galvanic corrosion, reducing the potential difference between the matrix and the second component, hence enhancing the material’s corrosion resistance. Moreover, the *in vitro* experimental results reveal that the ZX composite maintains a low corrosion rate and promotes robust bone tissue growth around the implant.

## Data Availability

The original contributions presented in the study are included in the article/Supplementary material, further inquiries can be directed to the corresponding authors.

## References

[B1] Abdel-Gawad S. A. Shoeib M. A. (2019). Corrosion studies and microstructure of Mg−Zn−Ca alloys for biomedical applications. Sur. Inter. 14, 108–116. 10.1016/j.surfin.2018.11.011

[B2] Ali M. Hussein M. A. Al-Aqeeli N. (2019). Magnesium-based composites and alloys for medical applications: A review of mechanical and corrosion properties. J. Alloy. Compd. 792, 1162–1190. 10.1016/j.jallcom.2019.04.080

[B3] Aung N. N. Zhou W. (2010). Effect of grain size and twins on corrosion behaviour of AZ31B magnesium alloy. Corros. Sci. 52, 589–594. 10.1016/j.corsci.2009.10.018

[B4] Bahmani A. Arthanari S. Shin K. S. (2019). Corrosion behavior of Mg-Mn-Ca alloy: Influences of Al, Sn and Zn. J. Magnes. Alloy. 7, 38–46. 10.1016/j.jma.2018.11.004

[B5] Bakhsheshi-Rad H. Abdul-Kadir M. Idris M. Farahany S. (2012). Relationship between the corrosion behavior and the thermal characteristics and microstructure of Mg-0.5Ca-xZn alloys. Corros. Sci. 64, 184–197. 10.1016/j.corsci.2012.07.015

[B6] Cai S. Lei T. Li N. Feng F. (2012). Effects of Zn on microstructure, mechanical properties and corrosion behavior of Mg-Zn alloys. Mater. Sci. Eng. C 32, 2570–2577. 10.1016/j.msec.2012.07.042

[B7] Chang Y. Tseng C. Chao C. Chen C. H. Lin S. Y. Du J. K. (2020). Mg-Zn-Ca alloys for hemostasis clips for vessel ligation: *In vitro* and *in vivo* studies of their degradation and response. Materials 13, 3039. 10.3390/ma13133039 32646030 PMC7372433

[B8] Chen Y. Ying T. Yang Y. Wang J. Zeng X. (2023). Regulating corrosion resistance of Mg alloys via promoting precipitation with trace Zr alloying. Corros. Sci. 216, 111106. 10.1016/j.corsci.2023.111106

[B9] Cubides Y. Zhao D. Nash L. Yadav D. Xie K. Karaman I. (2020). Effects of dynamic recrystallization and strain-induced dynamic precipitation on the corrosion behavior of partially recrystallized Mg-9Al-1Zn alloys. J. Magnes. Alloy. 8, 1016–1037. 10.1016/j.jma.2020.09.005

[B10] Esmaily M. Svensson J. E. Fajardo S. Birbilis N. Frankel G. Virtanen S. (2017). Fundamentals and advances in magnesium alloy corrosion. Prog. Mater. Sci. 89, 92–193. 10.1016/j.pmatsci.2017.04.011

[B11] Fan Z. Gao F. Wang Y. Wang S. Patel J. (2022). Grain refinement of Mg-alloys by native MgO particles: An overview. J. Magnes. Alloy. 10, 2919–2945. 10.1016/j.jma.2022.10.006

[B12] Fan Z. Y. Zuo Y. B. Jiang B. (2011). A new Technology for treating liquid metals with intensive melt shearing. Mater. Sci. Forum. 690, 141–144. 10.4028/www.scientific.net/MSF.690.141

[B13] Fu J. Du W. Liu K. Du X. Zhao C. Liang H. (2022). Effect of the Ca2Mg6Zn3 phase on the corrosion behavior of biodegradable Mg-4.0Zn-0.2Mn-xCa alloys in hank’s solution. Materials 15, 2079. 10.3390/ma15062079 35329527 PMC8955503

[B14] Goh C. S. Gupta M. Wei J. Lee L. (2007). Characterization of high performance Mg/MgO nanocomposites. J. Compos. Mater. 41, 2325–2335. 10.1177/0021998307075445

[B15] Gu X.-N. Li S.-S. Li X.-M. Fan Y. B. (2014). Magnesium based degradable biomaterials: A review. Front. Mater. Sci. 8, 200–218. 10.1007/s11706-014-0253-9

[B16] Holweg P. Berger L. Cihova M. Donohue N. Clement B. Schwarze U. (2020). A lean magnesium-zinc-calcium alloy ZX00 used for bone fracture stabilization in a large growing-animal model. Acta Biomater. 113, 646–659. 10.1016/j.actbio.2020.06.013 32553919

[B17] Jafari S. Raman R. K. S. Davies C. H. J. Hofstetter J. Uggowitzer P. J. Löffler J. F. (2017). Stress corrosion cracking and corrosion fatigue characterisation of MgZn1Ca0.3 (ZX10) in a simulated physiological environment. J. Mech. Behav. Biomed. 65, 634–643. 10.1016/j.jmbbm.2016.09.033 27741493

[B18] Jiang M. G. Xu C. Yan H. Fan G. Nakata T. Lao C. (2018). Unveiling the formation of basal texture variations based on twinning and dynamic recrystallization in AZ31 magnesium alloy during extrusion. Acta Mater 157, 53–71. 10.1016/j.actamat.2018.07.014

[B19] Jin Y. Blawert C. Yang H. Wiese B. Feyerabend F. Bohlen J. (2020). Microstructure-corrosion behaviour relationship of micro-alloyed Mg-0.5Zn alloy with the addition of Ca, Sr, Ag, in and Cu. Mater. Des. 195, 108980. 10.1016/j.matdes.2020.108980

[B20] Jung I. Sanjari M. Kim J. Yue S. (2015). Role of RE in the deformation and recrystallization of Mg alloy and a new alloy design concept for Mg-RE alloys. Scr. Mater 102, 1–6. 10.1016/j.scriptamat.2014.12.010

[B21] Li Y. Wen C. Mushahary D. Sravanthi R. Harishankar N. Pande G. (2012). Mg-Zr-Sr alloys as biodegradable implant materials. Acta Biomater. 8, 3177–3188. 10.1016/j.actbio.2012.04.028 22531570

[B22] Lin G. Liu D. Chen M. You C. Li Z. Wang Y. (2018). Preparation and characterization of biodegradable Mg-Zn-Ca/MgO nanocomposites for biomedical applications. Mater. Charact. 144, 120–130. 10.1016/j.matchar.2018.06.028

[B23] Liu Y. Liu X. Zhang Z. Farrell N. Chen D. Zheng Y. (2019). Comparative, real-time *in situ* monitoring of galvanic corrosion in Mg-Mg2Ca and Mg-MgZn2 couples in Hank’s solution. Corros. Sci. 161, 108185. 10.1016/j.corsci.2019.108185

[B24] Luo Y. Deng Y. Guan L. Ye L. Guo X. Luo A. (2020). Effect of grain size and crystal orientation on the corrosion behavior of as-extruded Mg-6Gd-2Y-0.2Zr alloy. Corros. Sci. 164, 108338. 10.1016/j.corsci.2019.108338

[B25] Lu Y. Bradshaw A. R. Chiu Y. L. Jones I. (2015). Effects of secondary phase and grain size on the corrosion of biodegradable Mg-Zn-Ca alloys. Mater. Sci. Eng. C 48, 480–486. 10.1016/j.msec.2014.12.049 25579949

[B26] Moreno L. Mohedano M. Mingo B. Arrabal R. Matykina E. (2019). Degradation behaviour of Mg0.6Ca and Mg0.6Ca2Ag alloys with bioactive plasma electrolytic oxidation coatings. Coatings 9, 383. 10.3390/coatings9060383

[B27] Nie K. Zhu Z. Munroe P. Deng K. Han J. (2020). Effect of extrusion speed on mixed grain microstructure and tensile properties of a Mg-2.9Zn-1.1Ca-0.5Mn nanocomposite reinforced by a low mass fraction of TiCp. Mater. Sci. Eng. A 796, 140223. 10.1016/j.msea.2020.140223

[B28] Paul S. Ramasamy P. Das M. Mandal D. Renk O. Calin M. (2020). New Mg-Ca-Zn amorphous alloys: Biocompatibility, wettability and mechanical properties. Materialia 12, 100799. 10.1016/j.mtla.2020.100799

[B29] Peng H. Gong Z. Zan R. Wang W. Yu H. Sun Y. (2022). Research on the degradation behaviors of biomedical Mg-2 wt.% Zn alloy under a biliary environment *in vitro* and *in vivo* . J. Magnes. Alloy. 10.1016/j.jma.2022.10.019

[B30] Schäublin R. E. Becker M. Cihova M. Gerstl S. Deiana D. Hébert C. (2022). Precipitation in lean Mg-Zn-Ca alloys. Acta Mater 239, 118223. 10.1016/j.actamat.2022.118223

[B31] Shuai C. Wang B. Yang Y. Peng S. Gao C. (2019). 3D honeycomb nanostructure-encapsulated magnesium alloys with superior corrosion resistance and mechanical properties. Compos. B. Eng. 162, 611–620. 10.1016/j.compositesb.2019.01.031

[B32] Song G. Atrens A. (2003). Understanding magnesium corrosion-A framework for improved alloy performance. Adv. Eng. Mater. 5, 837–858. 10.1002/adem.200310405

[B33] Staiger M. P. Pietak A. M. Huadmai J. Dias G. (2006). Magnesium and its alloys as orthopedic biomaterials: A review. Biomaterials 27, 1728–1734. 10.1016/j.biomaterials.2005.10.003 16246414

[B34] Sun L. Ma H. Guan C. Wang J. Zhang P. Jin P. (2022). Roles of the heat treatment on the Mg-Nd phases and corrosion mechanisms of Mg-4Nd alloy under sulfuric acid type acid rain. Corros. Sci. 208, 110610. 10.1016/j.corsci.2022.110610

[B35] Tang C. Lyu S. Zhao Z. Chen M. (2023). Effects of MgO nano particles on the mechanical properties and corrosion behavior of Mg-Zn-Ca alloy. Mater. Chem. Phys. 297, 127380. 10.1016/j.matchemphys.2023.127380

[B36] Tie D. Guan R. Liu H. Chen M. Ulasevich S. A. Skorb E. V. (2022). *In vivo* degradability and biocompatibility of a rho-formed Mg-Zn–Sr alloy for ureteral implantation. J. Magnes. Alloy. 10, 1631–1639. 10.1016/j.jma.2020.11.005

[B37] Tsakiris V. Tardei C. Clicinschi F. M. (2021). Biodegradable Mg alloys for orthopedic implants-A review. J. Magnesium Alloys 9, 1884–1905. 10.1016/j.jma.2021.06.024

[B38] Wang B. Guan S. Wang J. Wang L. Zhu S. (2011). Effects of Nd on microstructures and properties of extruded Mg-2Zn-0.46Y-xNd alloys for stent application. Mater. Sci. Eng. B 176, 1673–1678. 10.1016/j.mseb.2011.03.015

[B39] Zander D. Zumdick N. A. (2015). Influence of Ca and Zn on the microstructure and corrosion of biodegradable Mg-Ca-Zn alloys. Corros. Sci. 93, 222–233. 10.1016/j.corsci.2015.01.027

[B40] Zeng R. Qi W. Cui H. Zhang F. Li S. Q. Han E. H. (2015). *In vitro* corrosion of as-extruded Mg-Ca alloys-The influence of Ca concentration. Corros. Sci. 96, 23–31. 10.1016/j.corsci.2015.03.018

[B41] Zhang B. Wang Y. Geng L. Lu C. (2012). Effects of calcium on texture and mechanical properties of hot-extruded Mg-Zn-Ca alloys. Mater. Sci. Eng. A 539, 56–60. 10.1016/j.msea.2012.01.030

[B42] Zhang J. Jiang B. Yang Q. Huang D. Tang A. Pan F. (2020). Role of second phases on the corrosion resistance of Mg-Nd-Zr alloys. J. Alloy. Compd. 849, 156619. 10.1016/j.jallcom.2020.156619

[B43] Zhang Y. Kevorkov D. Bridier F. Medraj M. (2011). Experimental study of the Ca-Mg-Zn system using diffusion couples and key alloys. Sci. Technol. Adv. Mat. 12, 025003. 10.1088/1468-6996/12/2/025003 PMC5090483 27877385

[B44] Zhao M. Liu M. Song G. Atrens A. (2008). Influence of the β-phase morphology on the corrosion of the Mg alloy AZ91. Corros. Sci. 50, 1939–1953. 10.1016/j.corsci.2008.04.010

